# Association between red meat intake and diabetes: a cross-sectional analysis of a nationally representative sample of US adults (NHANES 2003–2016)

**DOI:** 10.1017/S0007114526106497

**Published:** 2026-06-14

**Authors:** Djibril M. Ba, Yue Zhang, Tian Qiu, Nazia Raja-Khan, Ariana Pichardo-Lowden, Xiang Gao, Vernon M. Chinchilli

**Affiliations:** 1 Department of Public Health Sciences, Penn State College of Medicine, Hershey, PA, USA; 2 Department of Medicine, Division of Endocrinology, Diabetes and Metabolism, Penn State Health Milton S. Hershey Medical Center, Hershey, PA, USA; 3 Department of Nutrition and Food Hygiene, School of Public Health, Institute of Nutrition, Fudan University, Shanghai 200032, People’s Republic of China

**Keywords:** NHANES, Red meat, Diabetes, USA, Nationally representative sample, Diet

## Abstract

Greater consumption of red meat has been linked to a higher risk of mortality and chronic diseases, including diabetes. We aim to examine the associations between total, processed and unprocessed red meat intake and diabetes and to evaluate the substitution effects of other protein sources for red meat on diabetes. This population-based cross-sectional study utilised data from the National Health and Nutrition Examination Survey (NHANES) 2003–2016. Diabetes was defined as a self-reported diagnosis by a physician or other health professional, having a fasting plasma glucose of 126 mg/dl or higher, an HbA1c level of 6·5 % or higher, or the use of antidiabetic drugs. Multivariable logistic regression models were conducted. The study included 34 737 adult participants (mean (sd) age of 45·8 (17·5) years) from NHANES 2003–2016. After adjusting for major confounders, compared with the first quintile, higher intakes of total, processed and unprocessed red meat were positively associated with higher odds of diabetes, with adjusted OR of 1·49 (95 % CI 1·22, 1·81), 1·47 (95 % CI 1·17, 1·84) and 1·24 (95 % CI 1·06, 1·44), respectively. The corresponding *P*-trend values were (< 0.001, 0.001, and 0.006). In this nationally representative sample of US adults, participants in the highest quintiles of total, processed and unprocessed red meat intake had higher odds of diabetes than those in the lowest quintile. Substituting 1 serving/d of dietary protein from foods of plant origin (including nuts, seeds, legumes and soya) for total, processed or unprocessed red meat was associated with 9 % to 14 % lower odds of diabetes.

According to the Centers for Disease Control and Prevention (CDC), diabetes constitutes a growing burden on the public’s health that affects 12 % of the US population (over 40 million people with diagnosed or undiagnosed diabetes), with approximately 1.5 million new cases diagnosed each year^(1)^. Diabetes is considered the eighth leading cause of death in the USA, and the risk of premature death among individuals with diabetes is two to three times that of individuals without diabetes^(2–4)^. Growing evidence suggests that a greater consumption of red meat and processed meat is associated with a higher risk of mortality^(5,6)^, chronic diseases, including CHD^(7)^, certain types of cancer^(8,9)^, diabetes^(10)^ and hypertension^(11)^. Several non-pharmacological alternatives have been suggested to prevent diabetes and manage hyperglycaemia and insulin levels, including weight loss, increased physical activity and consumption of healthy diets rich in fruits, vegetables, whole grains and legumes, and low in saturated fats and red meat^(12–15)^.

Findings from a previous study using three longitudinal cohorts of the US adult population, confirmed by a meta-analysis, showed that consumption of processed, unprocessed and total red meat was positively associated with the risk of type 2 diabetes^(16)^. Other previous studies indicated that a greater consumption of red meat was associated with a higher risk of diabetes^(17–21)^. Moreover, additional studies have suggested that red meat intake, including processed and unprocessed red meat, was associated with several biomarkers of metabolic alterations related to diabetes, including hyperglycaemia, insulin levels, HbA1c levels, and higher BMI^(22,23)^. The association between total, processed and unprocessed red meat intakes and diabetes remains understudied in a nationally representative sample of the US adult population, a country with among the highest processed and unprocessed red meat consumption, making it directly relevant to the US population and national health policies and guidelines^(24)^. The present study is among the first to utilise data from the National Health and Nutrition Examination Survey (NHANES) cycles, thereby providing a more generalisable perspective. In addition, our substitution analysis using partition models contributes to the existing literature by offering novel insights into the potential benefits of substituting dietary proteins from foods of plant origin for different types of red meat in the prevention of diabetes. The present study aims to examine the associations of total, unprocessed and processed red meat intake with diabetes and to evaluate the substitution effects of other protein sources for red meat with diabetes. In exploratory analyses, we also assessed whether associations varied by poverty: income ratio (PIR) and food insecurity status.

## Methods

### Study population

The NHANES programme is designed to collect nationally representative health-related information to assess diet, medical history and other health-related behaviours of the non-institutionalised US population^(25)^. The NHANES evolved from the previous National Health Examination Survey (NHES), which began in the 1960s. However, NHANES, as it is now known, was founded in the early 1970s by combining the NHES with a greater focus on nutrition and health^(26)^. The nutrition data is critical for setting nutritional standards for the population and monitoring trends in healthy and unhealthy eating habits^(27)^. The study population consisted of participants of the continuous NHANES 2003 to 2016, who completed a 24-h dietary recall. The NHANES is an ongoing series of nationally representative health surveys conducted by the National Center for Health Statistics of the CDC. The surveys use a multistage probability sampling design to develop a sample representative of the US population^(28)^. The NHANES data are publicly available through the CDC website^(29)^. The NHANES survey protocols are approved annually by the National Center for Health Statistics Research Ethics Review Board, and all participants provide written informed consent^(25)^. Since NHANES data are de-identified and publicly available, the Institutional Review Board at the researchers’ institution does not consider this to be human subject research. Therefore, further human subjects’ approval was unnecessary for the present study.

**Figure 1.**
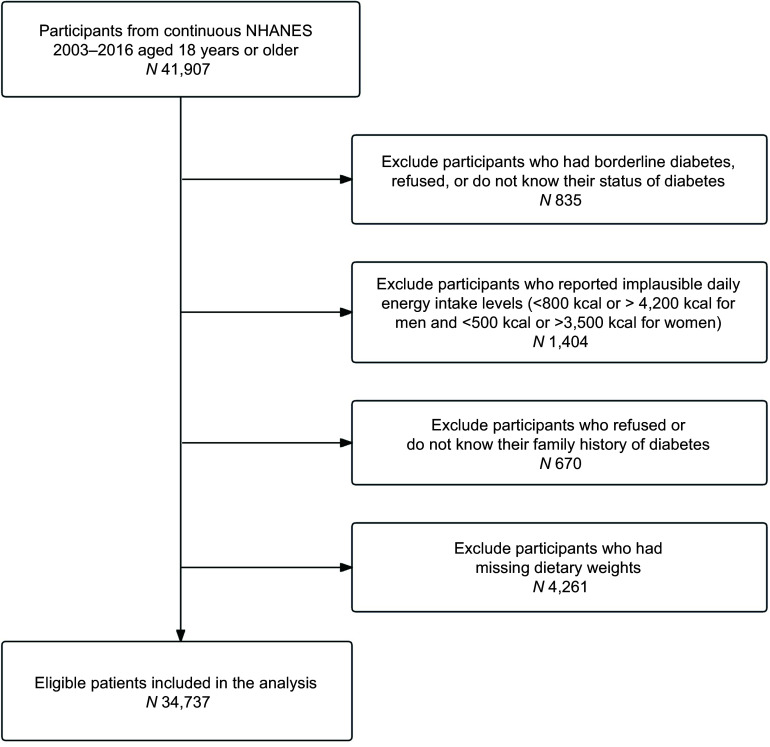
Flow chart of the study participants.

The present study included participants aged 18 years or older (*n* 41 907) from the NHANES 2003–2016 data. Participants were excluded if they had borderline diabetes, as defined by the NHANES questionnaire response option ‘Doctor told you have diabetes’, or if they refused to answer or did not know their diabetes status (*n* 835), reported implausible daily energy intake levels (< 800 kcal or > 4200 kcal for men and < 500 kcal or > 3500 kcal for women) (*n* 1404), refused or do not know their family history of diabetes (*n* 670) or had missing dietary sample weights (*n* 4261). A total of 34 737 participants were included in the present analysis (Figure 1).

### Assessment of diet

Participants who were examined during NHANES were also eligible to complete 24-h dietary recall interviews in which respondents reported all foods and beverages consumed in the previous 24 h. During NHANES 2003–2016, among adults aged 18 years and older, over 85 % completed both 24-h dietary recalls^(30)^. While not all eligible participants completed both recalls, more than 95 % completed at least one recall. On the first day (Day 1), dietary recall surveys were collected in person at the Mobile Examination Center by trained interviewers. The second day (Day 2) of dietary recall interviews was conducted by telephone, 3–10 days after the Mobile Examination Center interviews^(31)^. The NHANES 24-h dietary recalls were collected using the computerised method of the US *Department of Agriculture* (USDA) Automated Multiple-Pass Method, which accounts for day-to-day variation^(32)^. Unprocessed red meat includes beef, veal, pork, lamb and game meat. We used the MyPyramid Equivalents Database 2.0 for 2003–2004 and the USDA Food Patterns Equivalents Database for 2005–2016 to estimate food patterns, including red and processed meat intake. The USDA Food Patterns Equivalents Database is the updated version of the MyPyramid Equivalents Database. Red and processed meat intakes were directly obtained from these NHANES-specific databases. Food pattern components are reported in cup equivalents for fruits, vegetables and dairy products and in ounce equivalents for grains and protein foods^(33)^. Processed meat included frankfurters, sausage and luncheon meats. For this study, total red meat was defined as the combination of unprocessed red meat and processed meat. For participants with two 24-h dietary recalls (Day 1 and Day 2), dietary data were averaged; for those with only one recall, the single-day data were used.

### Ascertainment of diabetes

For the present study, diabetes was defined using any of the following criteria: self-report of diabetes diagnosis by a physician or other health professional, having a fasting plasma glucose level of 126 mg/dl or more, an HbA_1c_ level of 6·5 % or more, or use of antidiabetic drugs in the past 30 d prior to the survey interview^(34)^.

### Assessment of covariates

The following covariates were directly extracted from the NAHNES databases: sociodemographic factors including age (years), sex (men/women), ethnicity-race (Mexican American, other Hispanic, Non-Hispanic White, other race-multi-racial), education (less than high school, high school degree, more than high school), marital status (never married/married), BMI (kg/m^2^), food insecurity status (yes/no), PIR (≤ 1·30, > 1·30), psychological distress (yes/no), lifestyle factors including smoking status (non-smokers, former, current smokers), physical activity expressed as metabolic equivalent of task (MET) minutes per week, alcohol intake (g/d), high cholesterol, which was defined based on self-reported physician diagnosis of high cholesterol (yes/no), family history of diabetes (yes/no) and CVD (yes/no) were recorded as separate binary variables, and total calories intake (kcal/d) were included. Other dietary-related variables, including but not limited to the consumption of fish and shellfish (serving/d), poultry (serving/d), vegetables (serving/d), fruit (serving/d) and sugar-sweetened beverages (kcal/d) were also included in this analysis. During the Mobile Examination Center, weight and height were measured by trained technicians. BMI was calculated as weight (in kilograms) divided by height (in metres) squared. The PIR is the ratio of family income to the poverty threshold. It is calculated by dividing the family income by poverty guidelines specific to family size, year and state. Participants with a PIR ≤ 1·30 are considered to have low income (below 130 % of the federal poverty line), while those with a PIR > 1·30 are considered to have higher income (above 130 % of the federal poverty line)^(35)^. More than high school’ refers to individuals with some college or an associate degree, or those with a college degree or higher (bachelor’s degree or above). Psychological distress was defined as the use of anti-anxiety or antidepressant medications. NHANES measures of physical activity were self-reported using the Global Physical Activity Questionnaire. The total physical activity score (MET-minutes per week) was calculated by summing MET-minutes per week across all activity domains. This score was then categorised into three groups: < 600, 600–1199 and ≥ 1200 MET-minutes per week. Food security status was assessed using the Household Food Security Module developed by the USDA^(36)^. The module included eighteen items for households with children younger than 18 years and ten items for households without children. Food insecurity status was treated as a binary variable. Consistent with previous studies, participants who reported three or more affirmative responses were classified as food insecure (coded as ‘yes’), corresponding to low or very low food security; otherwise, responses were coded as ‘no’ (food secure)^(37)^.

### Statistical analysis

All analyses followed the National Center for Health Statistics guidelines, using appropriate weights to account for the complex sampling design of the NHANES survey, including strata, cluster and mobile examination centre weights^(38)^. To describe the characteristics of the study population, we performed univariable analyses. Imputation was performed with the hot-deck technique for participants with missing demographic and lifestyle variables using PROC SURVEYIMPUTE^(39)^. Data missingness ranged from 0·08 % for education level to 29·6 % for the physical activity variable. Distributions of all variables were examined before and after imputation, and the overall data structure was well preserved. Multivariable logistic regression models (PROC SURVEYLOGISTIC; SAS Institute) were used to examine the independent associations of total red meat, unprocessed and processed meat intake with diabetes, adjusting for potential confounders, including total energy intake, demographics, lifestyle factors and other dietary factors. We conducted a nutritional substitution analysis to compare the health impacts of substituting 1 serving/d of other protein sources, such as poultry, fish, eggs, dairy products and plant-based protein sources (nuts, seeds, legumes and soya) for total, processed and unprocessed red meat. To facilitate interpretation of the results and maintain consistency with previous studies, we combined all plant-based protein sources, as defined and reported in NHANES, into a single composite variable. Food substitution analyses complement overall dietary pattern analyses but do not replace them. However, substitution results are generally easier to interpret than analyses of single foods that do not specify a comparison. This distinction is important because increasing the intake of one food typically decreases the intake of another; therefore, the overall health impact depends on both foods^(40)^. The dietary components were originally expressed using USDA food patterns (cup equivalents for fruits, vegetables and dairy products; ounce equivalents for grains and protein foods)^(33)^. To improve interpretability, we converted these into servings/d using standardised factors (e.g. 1 serving of poultry, fish or soya = 3·5 oz; legumes = 2 oz; others, such as fruits and vegetables = 1 oz or 1 cup equivalent).

As in a previous study, one serving of unprocessed red meat equals 3 oz of beef, veal, pork, lamb or game; one serving of processed red meat equals 1·6 oz of sausages, frankfurters and luncheon meats made from beef, pork or poultry^(41)^.

The association of substituting 1 serving/d of protein sources for 1 serving/d of total, processed and unprocessed red meat with diabetes was assessed by including the alternative foods as continuous variables in the same multivariable logistic regression model after adjusting for total energy intake in addition to dietary and non-dietary factors^(42)^. The difference in the regression coefficients of the two foods being compared, and their variances and covariances, was used to estimate the *β* coefficient and variance for the substitution effect, which was used to calculate OR and 95 % CI for the substitution effect^(16,42–44)^. Linear trends were tested for significance using the median intake for each quintile of total, processed and unprocessed red meat, modelled as a single continuous variable. Effect modification was tested by including the multiplicative interaction term between total red meat intake and sex, age, PIR, psychological distress and food insecurity. We conducted a sensitivity analysis by excluding individuals classified as experiencing psychological distress. The multivariable logistic regression results were presented as adjusted OR and the 95 % CI. Data were analysed in SAS software (version 9.4; SAS Institute) at a two-tailed *α* level of 0·05.

## Results

We included a total of 34 737 participants (mean (sd) age of 45·8 (17·5) years) in the analysis, of whom 10·5 % had diabetes. Participants with the highest category of total red meat intake were more likely to be men (72·6 %), non-Hispanic White (69·4 %), younger, non-smokers (48·7 %), married (63·8 %), more educated (55·7 %) and physically more active; they also had higher alcohol intake, higher BMI and higher total calorie intake compared with participants in lower quintiles (Table 1). Approximately 14 % of the overall sample were food insecure, with food insecurity varying slightly across quintiles of total red meat intake but remaining generally similar overall (Table 1). Compared with the lower fifths of the distribution, participants in the highest fifth of total red meat intake reported a slightly higher prevalence of family history of diabetes and a lower prevalence of CVD history, except when compared with the lowest fifth (Table 1). Moreover, dairy products intake increased slightly across quintiles of total red meat intake. Although whole-grain intake was lower only relative to the bottom fifth, participants with higher total red meat consumption had lower intakes of fruit, fish, and poultry, plant-based proteins, and higher coffee consumption (Table 1).

**Table 1.** Baseline characteristics of participants (*n* 34 737) according to quintiles of total red meat intake based on NHANES 2003–2016 data Table 1 long description.

	Quintile of total red intakes^*^									
Characteristics	Q1 (*n* 6931)		Q2 (*n* 7075)		Q3 (*n* 7059)		Q4 (*n* 7022)		Q5 (*n* 6650)	
Median intake, oz/d	0·0		0·99		2·00		3·28		5·72	
IQR			0·75, 1·24		1·76, 2·29		2·92, 3·70		4·84, 7·23	
Age										
Mean	45·7		47·2		46·8		45·6		43·5	
sd	17·8		18·6		17·5		17·3		16·2	
	*n*	%	*n*	%	*n*	%	*n*	%	*n*	%
Women	4393	66·0	4534	64·4	4051	60	3259	45·3	1915	27·4
Non-Hispanic White	2692	63·7	3017	66·4	3208	68·8	3266	69·0	3103	69·4
Non-smokers	4382	62·4	4159	57·6	3928	55·1	3634	52·5	3210	48·7
More than high school	3637	63·7	3529	60·3	3535	59·4	3451	58·5	3094	55·7
Married	3918	59·6	4039	60·5	4229	64·8	4213	63·5	4116	63·8
Poverty:income ratio ≤ 1·30	2162	23·4	2235	23·0	2186	21·7	2165	20·7	2072	21·4
Psychological distress^†^	939	16·8	946	16·0	926	15·8	864	13·9	725	12·4
BMI (kg/m^2^)										
Mean	27·7		28·3		28·7		29·1		29·2	
sd	6·5		6·6		6·8		7·0		6·8	
Physical activity (≥ 1200 MET-min/week)	4291	63·4	4235	61·4	4200	60·5	4406	62·9	4293	65·0
High cholesterol	2489	33·3	2759	38·1	2652	36·5	2623	36·0	2470	36·7
Family history of diabetes	2731	36·3	2871	37·5	2882	38·0	2884	38·9	2740	39·0
History of CVD	683	7·1	805	9·4	810	8·9	796	9·0	638	7·7
Food insecure	1305	13·8	1282	13·8	1305	14·0	1284	13·5	1272	14·3
	Mean	sd	Mean	sd	Mean	sd	Mean	sd	Mean	sd
Alcohol (g/d)	7·2	17·7	7·0	17·1	8·1	19·6	9·5	22·3	12·8	25·0
Total energy (kcal/d)	1782·8	663·0	1846·0	648·2	1958·1	644·2	2159·2	684·0	2503·2	713·1
Fruit (serving/d)	0·9	1·1	0·7	0·9	0·7	0·9	0·7	0·9	0·6	0·9
Vegetable (serving/d)	0·7	0·8	0·6	0·6	0·6	0·6	0·6	0·6	0·7	0·7
Plant-based proteins (serving/d)	1·1	1·8	0·8	0·6	0·9	1·5	0·8	1·5	0·8	1·5
Fish (serving/d)	0·3	0·6	0·2	0·4	0·2	0·4	0·1	0·4	0·1	0·4
Poultry (serving/d)	0·6	0·7	0·5	0·6	0·4	0·5	0·4	0·5	0·3	0·5
Dairy products (serving/d)	1·4	1·3	1·5	1·2	1·5	1·2	1·6	1·2	1·7	1·4
Egg (serving/d)	0·5	0·7	0·5	0·7	0·5	0·7	0·5	0·7	0·6	0·8
Whole grains (serving/d)	1·0	1·3	0·8	1·0	0·8	1·0	0·8	1·1	0·8	1·1
SSB (kcal/d)	102·1	170·6	117·9	174·1	127·9	182·1	154·6	202·7	177·0	224·0
Coffee (g/d)	275·2	385·2	278·0	392·6	299·7	422·9	304·3	430·4	323·0	463·9

SSB, sugar-sweetened beverages.

* Unweighted sample size (*n*); weighted mean (standard deviation) for continuous variable, and weighted proportion for categorical variable.

† Psychological distress was defined as the use of anti-anxiety or antidepressant medications; plant-based proteins (nuts, seeds, legumes and soya).

In this study, and after adjustment for major confounders including demographics, lifestyle factors, psychological distress, diet quality including intake of fruits and vegetables, sugar-sweetened beverages, coffee, whole grains, and major protein food sources such as poultry, eggs, dairy products, legumes, nuts, and seeds, and BMI (Model 4), intakes of total red meat, processed red meat and unprocessed red meat were all associated with higher odds of diabetes (Table 2). Participants in the highest quintile of total red meat, compared with those in the lowest quintile, had 49 % higher odds of diabetes (OR_Q5 *v*. Q1_ = 1·49, 95 % CI 1·22, 1·81; *P*-trend < 0·001). Comparing the highest quintiles, processed red meat intake was also associated with 47 % higher odds of diabetes (OR_Q5 *v*. Q1_ = 1·47, 95 % CI 1·17, 1·84; *P*-trend = 0·001), whereas unprocessed red meat intake was associated with 24 % higher odds of diabetes (OR_Q5 *v*. Q1_ = 1·24, 95 % CI 1·06, 1·44; *P*-trend = 0·006) (Table 2).

**Table 2.** Associations between total, processed and unprocessed red meat intake and diabetes in NHANES 2003–2016, showing the odds ratios (ORs) and 95% confidence intervals (CIs) Table 2 long description.

	Quintiles of red meat intakes^*^											
Total red meat intake	Q1	Q2		Q3		Q4		Q5		*P*-trend	OR per serving/d	95 % CI
Median intake (IQR), oz/d	0·0	0·99	(0·75–1·24)	2·00	(1·76–2·29)	3·28	(2·92–3·70)	5·72	(4·84–7·23)			
No. of cases	919	1036		1018		1038		919				
Model 1	1(ref)	1·11	0·94–1·30	1·16	0·99–1·36	1·37	1·18–1·59	1·41	1·19–1·67	< 0·001	1·13	1·06, 1·20
Model 2	1(ref)	1·11	0·93–1·33	1·24	1·05–1·47	1·50	1·28–1·76	1·64	1·35–2·00	< 0·001	1·21	1·13, 1·30
Model 3	1(ref)	1·11	0·92–1·34	1·27	1·07–1·49	1·55	1·32–1·83	1·77	1·45–2·16	< 0·001	1·26	1·16, 1·36
Model 4	1(ref)	1·10	0·90–1·33	1·20	1·01–1·42	1·38	1·15–1·67	1·49	1·22–1·81	< 0·001	1·16	1·07, 1·25
Processed red meat intake												
Median intake (IQR), oz/d	0·0	0·23	0·12–0·35	0·76	0·57–0·90	1·48	1·21–1·67	2·96	2·36–3·89			
No. of cases	1717	755		828		792		838				
Model 1	1(ref)	0·98	0·84–1·14	1·20	1·00–1·44	1·02	0·87–1·20	1·51	1·23–1·85	< 0·001	1·15	1·08, 1·22
Model 2	1(ref)	0·96	0·81–1·14	1·21	0·98–1·48	1·05	0·88–1·26	1·59	1·26–2·01	< 0·001	1·18	1·10, 1·26
Model 3	1(ref)	1·02	0·86–1·20	1·22	0·99–1·50	1·06	0·89–1·27	1·62	1·29–2·04	< 0·001	1·18	1·11, 1·26
Model 4	1(ref)	1·06	0·90–1·25	1·25	1·01–1·54	1·02	0·86–1·22	1·47	1·17–1·84	0·001	1·10	1·03, 1·19
Unprocessed red meat intake												
Median intake (IQR), oz/d	0·0	0·30	0·14–0·45	1·05	0·81–1·27	2·13	1·79–2·46	4·31	3·50–5·64			
No. of cases	1466	486		1082		994		902				
Model 1	1(ref)	0·96	0·81–1·13	1·12	0·97–1·31	1·15	0·99–1·34	1·12	0·97–1·28	0·07	1·05	0·98, 1·12
Model 2	1(ref)	0·95	0·79–1·14	1·17	0·99–1·37	1·22	1·04–1·43	1·26	1·08–1·47	0·002	1·12	1·04, 1·20
Model 3	1(ref)	0·96	0·80–1·14	1·19	1·01–1·40	1·25	1·07–1·46	1·34	1·16–1·56	< 0·001	1·15	1·07, 1·24
Model 4	1(ref)	0·98	0·82–1·17	1·22	1·03–1·44	1·20	1·03–1·40	1·24	1·06–1·44	0·006	1·10	1·02, 1·19

* Dietary intake was collected using 24-h dietary recalls. One serving of unprocessed red meat equals 3 oz of beef, veal, pork, lamb or game meat; one serving of processed red meat equals 1·6 oz of sausages, frankfurters or luncheon meats made from beef, pork or poultry.

Model 1: Adjusted for age and sex.

Model 2: Further adjusted for model 1 covariates plus smoking status, alcohol intake, race/ethnicity, education, family poverty:income ratio, marital status, physical activity, family history of diabetes, high cholesterol, history of CVD, total energy intake, food insecurity status and survey year.

Model 3: Further adjusted for model 2 covariates plus dietary intakes of fruits and vegetables, poultry, fish, eggs, dairy products, plant-based protein sources (nuts, seeds, legumes and soya), whole grains and coffee.

Model 4: Further adjusted for model 3 covariates plus sugar-sweetened beverage (SSB) intake, psychological distress and BMI.

In continuous analyses (model 4), every 1 serving/d increment in total red meat was associated with 1·16 times higher odds of diabetes (95 % CI 1·07, 1·25) (Table 2). Moreover, every 1 serving/d increment of processed red meat was associated with 1·10 times higher odds of diabetes (95 % CI 1·03, 1·19), and that increment of unprocessed red meat was associated with 1·10 times higher odds of diabetes (95 % CI 1·02, 1·19) (Table 2).

In our exploratory analysis, we did not observe significant interactions between continuous total red meat, unprocessed red meat or processed red meat intake and PIR, or between these red meat types and food insecurity status (*P*-interaction > 0·05 for all).

Furthermore, we did not observe any significant interaction between total red meat intake and sex, age, psychological distress or food insecurity status (*P*-interaction > 0·05, for all).

**Figure 2.**
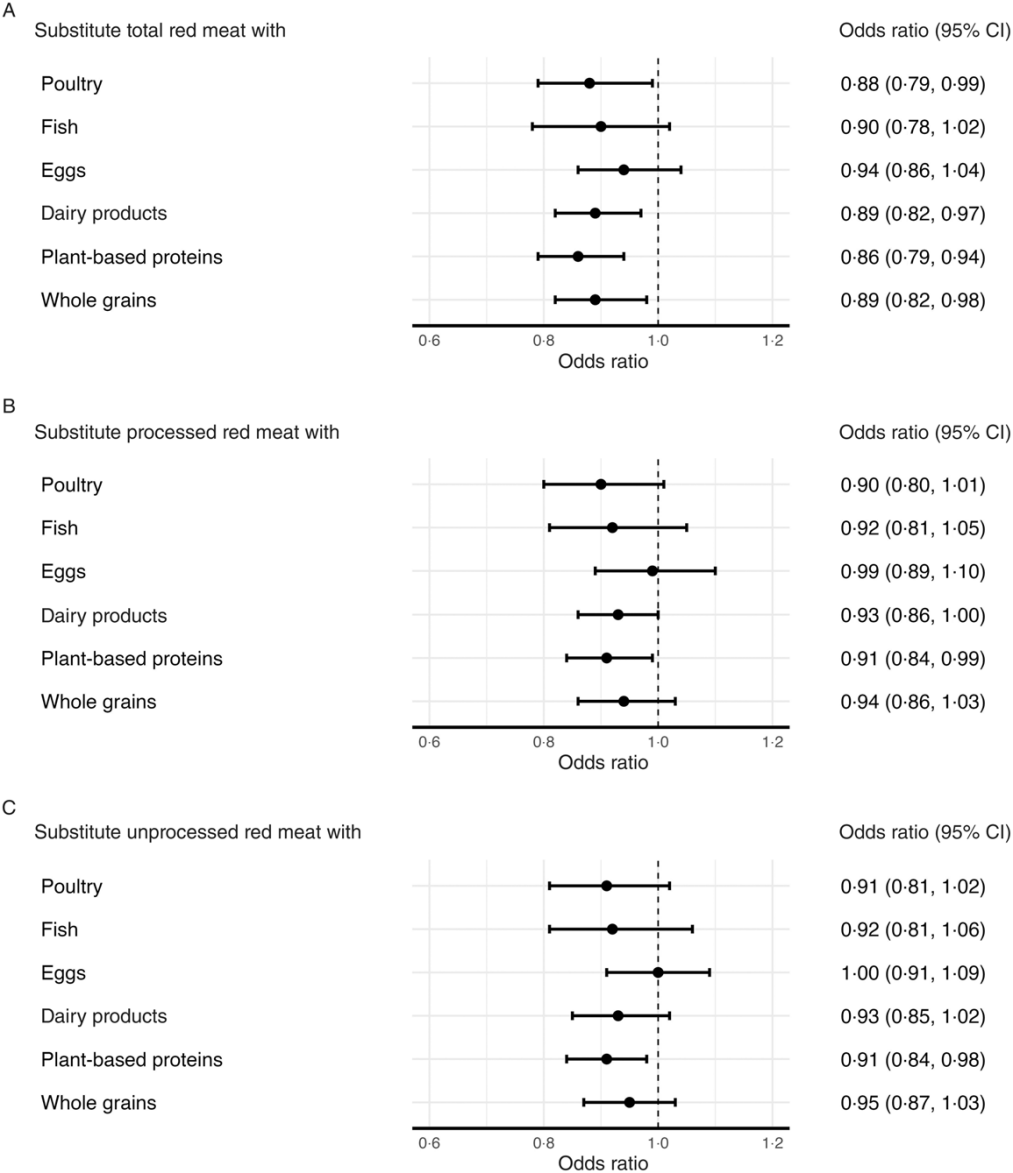
Associations between substituting one serving of other protein sources for red meat intake and the odds of diabetes in the NHANES (2003–2016). The models were adjusted for age, sex, smoking status, alcohol intake, race/ethnicity, education level, poverty: income ratio, marital status, physical activity, family history of diabetes, high cholesterol, history of CVD, total energy intake, food insecurity status, survey year, dietary intakes of fruits and vegetables, sugar-sweetened beverages (SSB), psychological distress and BMI, excluding the foods being substituted.

In substitution analyses using 1 serving/d of plant-based protein sources as the replacement, replacing 1 serving/d of total red meat was associated with 14 % lower odds of diabetes (OR = 0·86, 95 % CI 0·79, 0·94, Figure 2, Panel A). Similarly, substitutions of 1 serving/d of poultry, dairy products and whole grains for total red meat were associated with 11–12 % lower odds of diabetes (poultry: OR = 0·88, 95 % CI 0·79, 0·99; dairy products: OR = 0·89, 95 % CI 0·82, 0·97; whole grains: OR = 0·89, 95 % CI 0·82, 0·98, Figure 2, Panel A). For processed red meat, substituting 1 serving/d of plant-based protein sources for 1 serving/d of processed red meat was associated with 9 % lower odds of diabetes (OR = 0·91, 95 % CI 0·84, 0·99; Figure 2, Panel B). Similarly, replacing 1 serving/d of unprocessed red meat with 1 serving/d of plant-based protein sources was associated with 9 % lower odds of diabetes (OR = 0·91, 95 % CI 0·84, 0·98, Figure 2, Panel C).

## Discussion

In this large, nationally representative, unweighted sample of 34 747 US adults using NHANES data from 2003 to 2016, the overall weighted prevalence of diabetes was 10·5 % (95 % CI 9·97, 11·09). After adjustment for sociodemographic, lifestyle and dietary factors, higher intakes of total red meat, processed red meat and unprocessed red meat were all positively associated with higher odds of diabetes. More importantly, the findings were consistent after adjusting for BMI, suggesting that the observed associations were independent of BMI. Consistent with previous prospective cohort studies, all observed associations were significant, but the association was stronger for processed meat than for unprocessed red meat^(12,16,41)^. In addition, excluding participants experiencing psychological distress did not alter the observed associations for different types of red meat intake. Our substitution analyses indicate that substituting red meat with alternative protein sources is associated with lower odds of diabetes. Specifically, substituting total red meat with poultry, dairy products, whole grains or plant-based protein sources was associated with 11–14 % lower odds, with plant-based protein sources showing the strongest association. For both processed and unprocessed red meat, replacing 1 serving/d with plant-based proteins was consistently associated with 9 % lower odds of diabetes, underscoring the benefit of limiting red meat intake and reinforcing the importance of alternative protein sources for diabetes prevention.

To the best of our knowledge, this study is among the first studies to examine the association between red meat intake and diabetes using a nationally representative sample of the US adult population. Furthermore, our food substitution analyses serve to complement, rather than replace, overall dietary pattern analyses. Substitution models are generally easier to interpret than analyses of single foods that do not specify a comparison. This distinction is important, as increasing the intake of one food necessarily decreases the intake of another; thus, the overall health impact depends on both foods^(40)^.

Our findings are directly generalisable to the US population in terms of national policies and public health relevance. Findings from a previous study using three longitudinal cohorts with a comprehensive dietary assessment using a validated FFQ, confirmed by a meta-analysis, as well as a recent study using Nurses’ Health Study, II, and Health Professionals Follow-up Study showed that greater intakes of total processed and unprocessed red meat were positively associated with the risk of type 2 diabetes^(16,41)^. Other previous studies indicated that a greater consumption of red meat is associated with a higher risk of diabetes^(17–21)^. A recent study, the Nurses’ Health Study II and the Health Professionals Follow-up Study, also indicated that intake of red meat, including both processed and unprocessed red meat, was strongly associated with a higher risk of type 2 diabetes^(41)^. A previous study using the European Prospective Investigation into Cancer and Nutrition data indicated a positive association between high consumption of total red meat and incident type 2 diabetes^(45)^. Moreover, other studies have suggested that red meat consumption is associated with higher fasting blood glucose, insulin concentrations and HbA1c levels^(22,23)^.

The higher content of saturated fats and heme iron^(46,47)^ in red meat, in addition to the high levels of Na^(48)^ in processed meat, may contribute to adverse health effects and increase the risk of diabetes. These dietary components are known to be positively associated with endothelial dysfunction, inflammation, insulin resistance and oxidative stress, which can cause tissue damage, especially reduced pancreatic *β*-cell function, thereby providing a plausible biological mechanism for the observed associations^(46,47,49–51)^.

The higher content of nitrates and Na in processed meat may contribute to adverse health effects, such as insulin resistance, and help explain the strongest relationship between red meat consumption and diabetes observed in the present study^(19)^. Findings from our substitution analyses are consistent with previous studies and suggest that alternative major plant-based protein sources, such as nuts and legumes, fish and eggs, were associated with the most substantial lower odds of diabetes^(12,41)^.

### Limitations

Our study, however, has some limitations that need to be addressed. First, diet was assessed using up to two 24-h dietary recalls, which may not have adequately captured within-person variation in total, processed and unprocessed red meat intake. In this context, random within-person error likely attenuated the point estimates and reduced the precision of the observed associations in the present study. In addition, the FPED does not disaggregate processed meat into processed red and processed poultry, which may result in non-differential misclassification of exposure. Second, because of its cross-sectional design, reverse causality emerging from changes in diet due to recent symptoms is possible. Moreover, the cross-sectional nature of the study limits our ability to assess causality beyond observed associations. Third, as in any observational study, residual confounding may be present. However, we carefully controlled for a set of major potential confounders, including sociodemographics, major lifestyle factors and other dietary risk factors, including total energy intake. As mentioned earlier, this study supports previous findings, highlighting that the focus is on higher *v*. lower red meat consumption rather than red meat intake *per se*. Therefore, our findings should be interpreted with caution. Lastly, because of the NHANES diabetes questionnaire’s structure, we were unable to differentiate between type 1 diabetes and type 2 diabetes in this study.

### Conclusions

In this study, greater intakes of total, processed and unprocessed red meat were associated with higher odds of diabetes, adding to the evidence of potential health concerns associated with higher red meat consumption compared with lower intake. Consistent with previous studies, substitution of alternative dietary components, particularly plant-based protein sources, poultry, dairy products, eggs or whole grains for total, processed or unprocessed red meat was associated with lower odds of diabetes in this study. These findings underscore the potential benefits of replacing red meat with alternative protein sources for diabetes prevention.

## Table 1 Long description

The table presents baseline characteristics of participants according to quintiles of total red meat intake. It includes data on median intake, age, gender, race, smoking status, education, marital status, poverty ratio, psychological distress, BMI, physical activity, cholesterol levels, family history of diabetes, history of CVD, food insecurity, alcohol intake, total energy intake, and various food servings. The table is divided into five quintiles (Q1 to Q5) with corresponding values for each characteristic. Each row represents a different characteristic, and each column represents a quintile of total red meat intake. Notable trends include higher alcohol intake, BMI, and total calorie intake in participants with the highest red meat intake. Additionally, participants in the highest quintile are more likely to be men, non-Hispanic White, non-smokers, married, more educated, and physically active. Food insecurity varies slightly across quintiles but remains generally similar. Participants in the highest quintile also report a slightly higher prevalence of family history of diabetes and a lower prevalence of CVD history. Dairy product intake increases slightly across quintiles, while whole-grain intake is lower only relative to the bottom quintile. Participants with higher red meat consumption have lower intakes of fruit, fish, poultry, plant-based proteins, and higher coffee consumption.

Navigate back to Table 1.

## Table 2 Long description

The table presents data on the associations between total, processed, and unprocessed red meat intake and diabetes, showing odds ratios (ORs) and 95% confidence intervals (CIs). The table is divided into three main sections: total red meat intake, processed red meat intake, and unprocessed red meat intake. Each section includes median intake in ounces per day, number of cases, and four models of odds ratios with confidence intervals. Row 1: Total red meat intake, Median intake (IQR), oz/d, 0.0, 0.99 (0.75-1.24), 2.00 (1.76-2.29), 3.28 (2.92-3.70), 5.72 (4.84-7.23). Row 2: No. of cases, 919, 1036, 1018, 1038, 919. Row 3: Model 1, I(ref), 1.11, 1.16, 1.37, 1.41 (P-trend < 0.001). Row 4: Model 2, I(ref), 1.11, 1.16, 1.37, 1.41 (P-trend < 0.001). Row 5: Model 3, I(ref), 1.11, 1.16, 1.37, 1.41 (P-trend < 0.001). Row 6: Model 4, I(ref), 1.10, 1.20, 1.38, 1.49 (P-trend < 0.001). Row 7: Processed red meat intake, Median intake (IQR), oz/d, 0.0, 0.23, 0.76, 1.48, 2.96. Row 8: No. of cases, 1717, 755, 828, 792, 838. Row 9: Model 1, I(ref), 0.98, 1.20, 1.02, 1.51 (P-trend < 0.001). Row 10: Model 2, I(ref), 0.96, 1.21, 1.05, 1.59 (P-trend < 0.001). Row 11: Model 3, I(ref), 1.02, 1.22, 1.06, 1.62 (P-trend < 0.001). Row 12: Model 4, I(ref), 1.06, 1.25, 1.02, 1.47 (P-trend 0.001). Row 13: Unprocessed red meat intake, Median intake (IQR), oz/d, 0.0, 0.30, 1.05, 2.13, 4.31. Row 14: No. of cases, 1466, 486, 1082, 994, 902. Row 15: Model 1, I(ref), 0.96, 1.12, 1.15, 1.12 (P-trend 0.002). Row 16: Model 2, I(ref), 0.95, 1.17, 1.22, 1.26 (P-trend < 0.001). Row 17: Model 3, I(ref), 0.96, 1.19, 1.25, 1.34 (P-trend < 0.001). Row 18: Model 4, I(ref), 0.98, 1.22, 1.20, 1.24 (P-trend 0.006).

Navigate back to Table 2.
